# Beyond the Bruise: A Radiological Journey Into Morel-Lavallée Lesions Through Four Illustrative Cases

**DOI:** 10.7759/cureus.66110

**Published:** 2024-08-04

**Authors:** Sakthi Ganesh Subramonian, Abdul Majith Seeni Mohamed, Seetha Rashi, Sukumar Ramaswami, Karthik Krishna Ramakrishnan

**Affiliations:** 1 Department of Radiology, Saveetha Medical College and Hospitals, Saveetha Institute of Medical and Technical Sciences, Saveetha University, Chennai, IND; 2 Department of Interventional Radiology, Saveetha Medical College and Hospitals, Saveetha Institute of Medical and Technical Sciences, Saveetha University, Chennai, IND

**Keywords:** compression therapy, pelvic trauma, sclerodesis, subcutaneous tissue separation, hemolymphatic collections, closed degloving injuries, morel-lavallée lesions

## Abstract

Morel-Lavallée lesions (MLLs) are rare closed degloving injuries resulting from significant trauma. These lesions occur when a high-impact force causes separation of the skin and subcutaneous tissue from the underlying fascia, leading to hemolymphatic collections. Despite their clinical significance, MLLs are frequently underdiagnosed, often leading to improper management and recurrence. This case series explores four illustrative cases of MLLs, highlighting the critical role of MRI in accurate diagnosis and staging. Detailed imaging features and optimal treatment options are discussed to guide clinicians in providing the best possible care. By raising awareness and providing a comprehensive understanding of these lesions, this series aims to enhance early detection and appropriate intervention, ultimately improving patient outcomes and reducing the mental distress associated with recurrent lesions.

## Introduction

Morel-Lavallée lesions (MLLs), first identified by the French physician François Morel-Lavallée in 1853, are closed degloving injuries that often result from significant trauma, leading to hemolymphatic mass or collection formations [1]. These lesions occur when a strong impact or compression causes the skin and subcutaneous fatty tissue to forcefully separate from the underlying fascia [1-3]. This happens along the interfascial planes between the fascia and muscles, creating a space that may fill with various fluids, including serous fluid or blood [4]. The fluid accumulation in these lesions can either resolve naturally or become chronic, forming an encapsulated collection [2,5,6]. Primarily occurring in the thigh, MLLs can also develop in areas such as the lumbar region, over the scapula, or near the knee due to similar biomechanical forces [1,7]. These lesions can be detected shortly after the trauma but may also present months or even years later in up to one-third of patients [8]. While CT scans are often used initially due to their quick availability in emergency situations, MRI is the preferred method for detailed imaging [9,10]. In cases where the lesions become chronic and encapsulated, conservative treatments such as compression bandages are usually ineffective [6-8]. Surgical intervention, including drainage or even capsule resection, may be necessary to prevent fluid reaccumulation [10].

## Case presentation

Case 1

Case 1 was a 24-year-old male patient with no significant past medical history who presented to the Emergency Department following a slip and fall from a train while intoxicated. He reported a sudden onset of worsening pain and swelling in the right hip area, along with multiple abrasions across his body. Upon examination, all vital signs were stable, and he was fully alert with a Glasgow Coma Scale score of 15. Cardiovascular and respiratory examinations were normal, including normal heart sounds and vesicular breath sounds. Abdominal examination showed no abnormalities. Neurologically, he was intact except for difficulty moving his right lower limb, suggesting a possible fracture.

**Figure 1:**
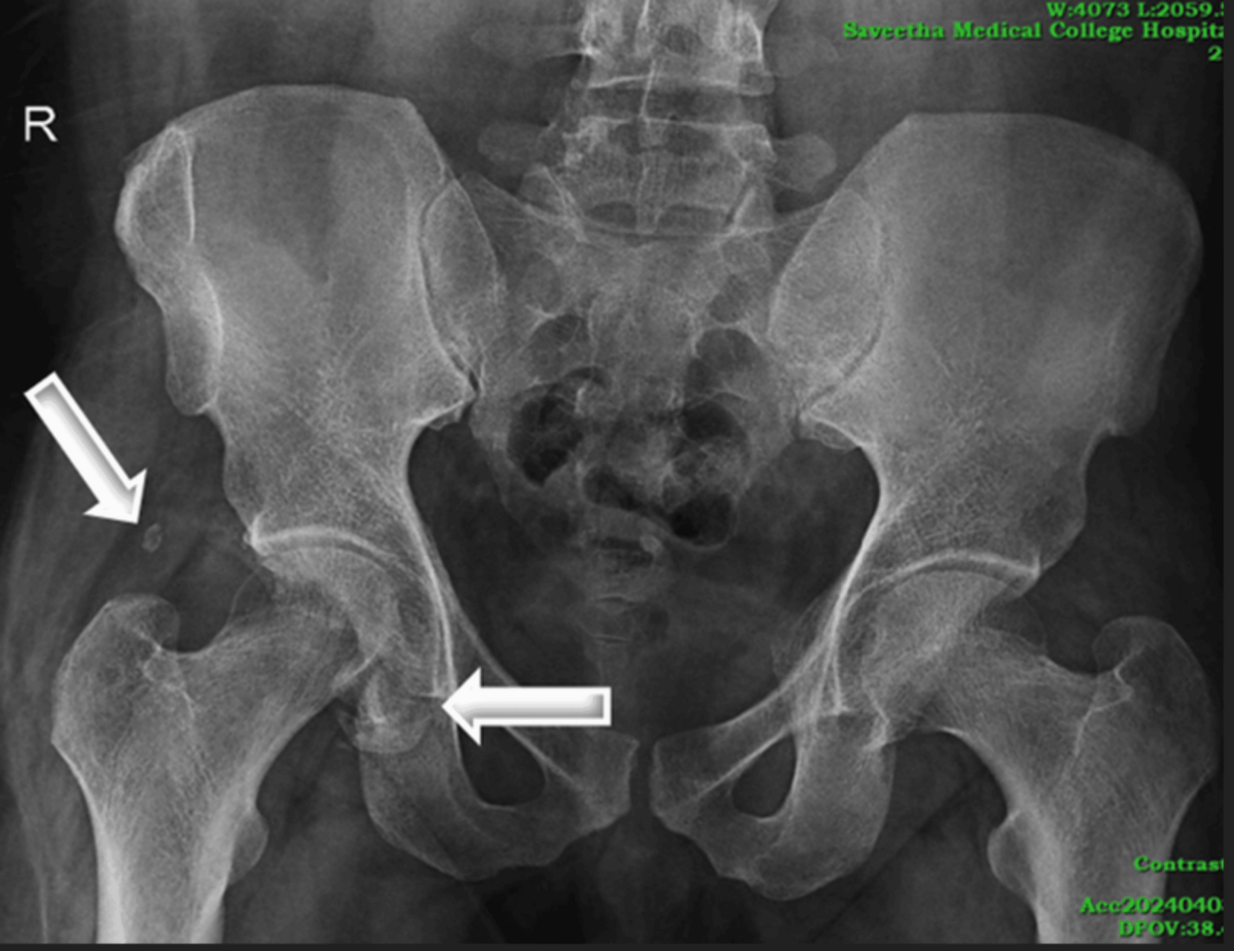
X-ray pelvis antero-posterior view showing mild disruption of Shenton's line with marked flattening of the infero-medial surface of the right femoral head with the fracture fragment displaced inferiorly and a radio-opacity noted over the greater trochanter of the right femur (likely bone fragment) represented by white arrows.

Initial X-ray of the pelvis revealed a mild disruption of Shenton's line, marked flattening of the infero-medial surface of the right femoral head with the fracture fragment displaced inferiorly, and a radio-opacity over the greater trochanter of the right femur, as shown in Figure 1.

**Figure 2:**
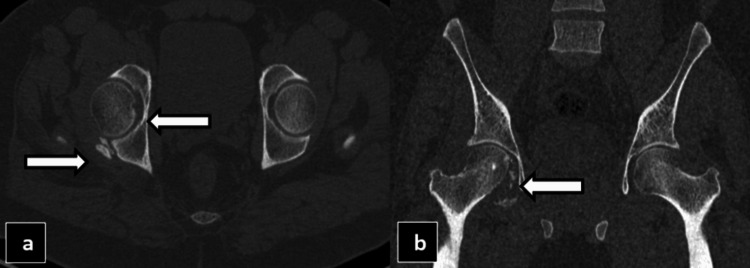
The CT pelvis axial reformatted image (a) and coronal reformatted image (b) in the bone window show comminuted displaced fractures involving the medial aspect of the articular surface of the right head of the femur with multiple adjacent bony fragments and comminuted fracture seen involving the posterior wall and posterior column of the right acetabulum (white arrows).

A subsequent CT scan revealed comminuted displaced fractures of the medial aspect of the right femoral head with multiple bony fragments; the largest fragment was inferiorly displaced, as shown in Figure 2. There was also a comminuted fracture involving the posterior wall and column of the right acetabulum, with the largest bony fragment displaced superiorly into the intramuscular plane, accompanied by a hematoma and fat stranding. The patient was initially managed conservatively.

**Figure 3:**
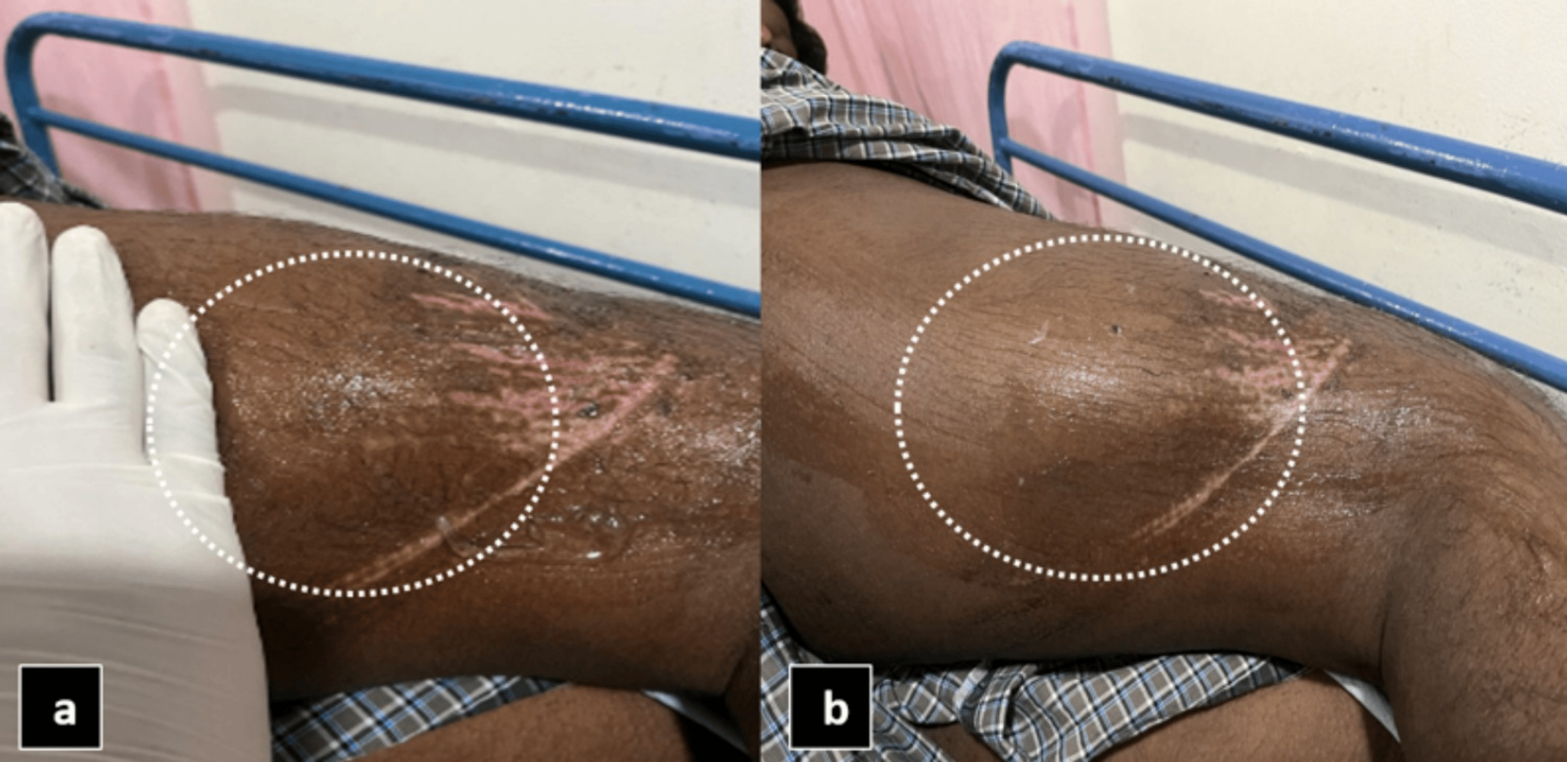
Gross images (a and b) of the swelling in the lateral aspect of the right thigh, represented by the dotted circle.

One week post-injury, the patient developed swelling in the lateral aspect of the right thigh, as shown in Figure 3 and Video 1.

**Video 1:** Video demonstrating the fluctuant swelling located on the lateral aspect of the patient's right thigh.

**Figure 4:**
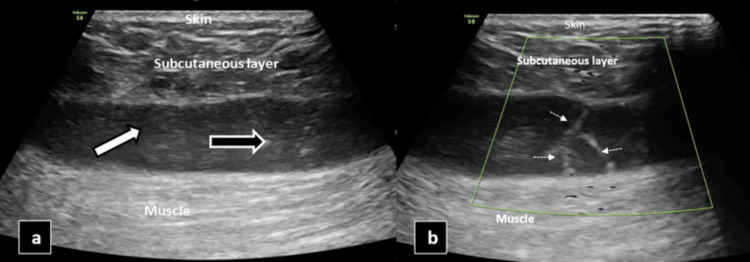
USG superficial image of the right thigh (a) showing anechoic fluid collection between the subcutaneous layer and muscle by the white arrow and a few internal echoes likely fat lobules represented by the black arrow. USG color Doppler (b) shows multiple tiny linear hyperechoic internal septations with no evidence of internal vascularity likely avascular septae represented by white dotted arrows.

An ultrasonogram was conducted in the region of interest (right thigh) revealing an anechoic fluid collection of volume ~50cc with few internal echoes (likely fat lobules) between the subcutaneous and fascial layers in the affected area, and color Doppler showed multiple tiny avascular septae, as shown in Figure 4.

Case 2

**Figure 5:**
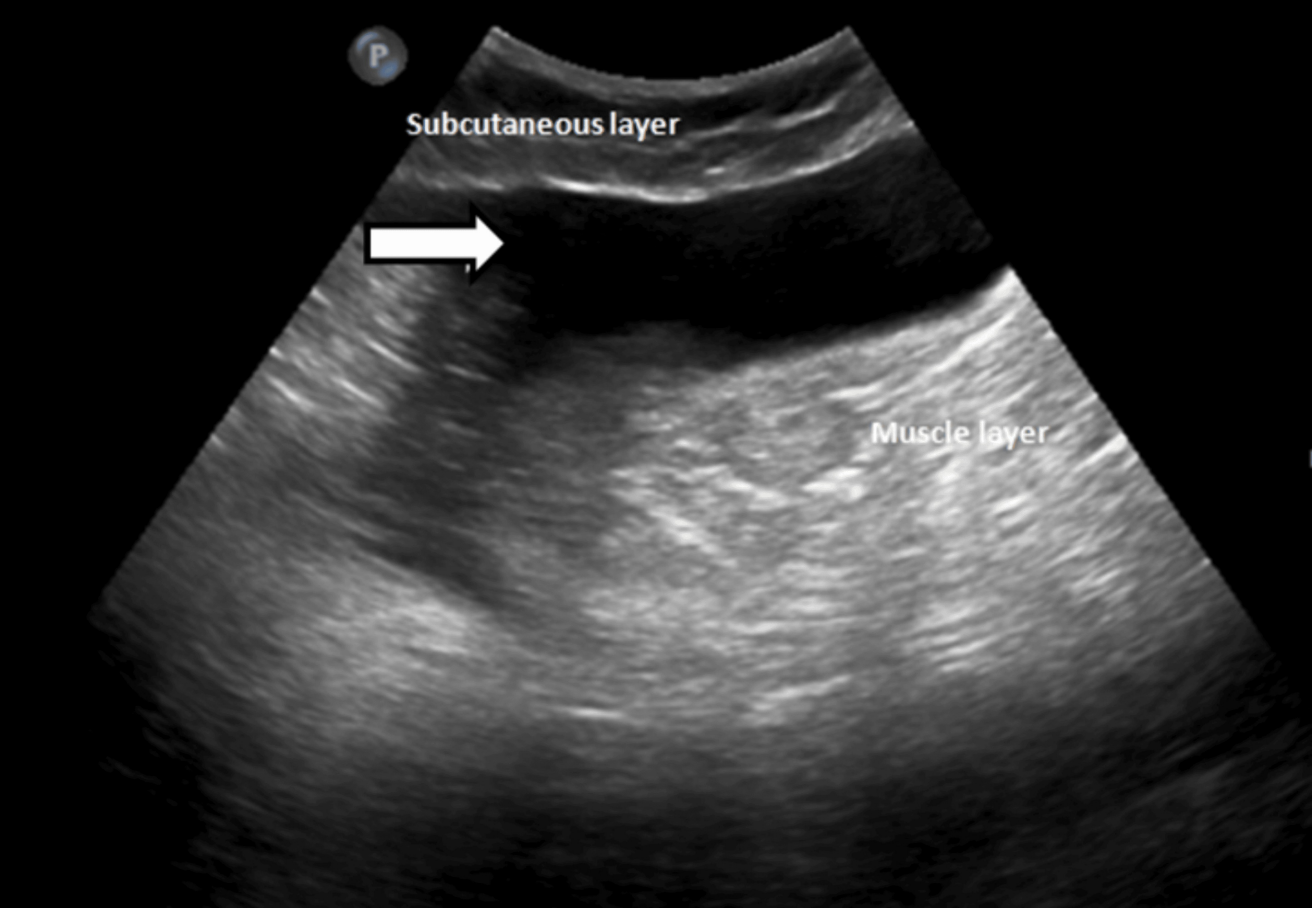
Ultrasound image showing an anechoic fluid collection of volume ~80-90cc noted between the subcutaneous layer and muscle (white arrow) of the right thigh (region of interest).

In Case 2, a 26-year-old female patient presented to the Interventional Radiology Department with chief complaints of swelling and a postoperative fluid collection in the right loin area. She has a significant past medical history, being a survivor of a road traffic accident (RTA) one year ago, which resulted in multiple traumas. She sustained fractures to the pelvic bones and lumbar vertebrae and suffered extensive lacerations and abrasions across her body. Additionally, there was a raw area in the right thigh region that became infected subsequent to the accident. To manage the infection, debridement and split-thickness skin grafting were performed on the affected area. A recent screening ultrasound was conducted, revealing an anechoic fluid collection of approximately 80-90cc located between the subcutaneous and muscular planes (Figure 5).

**Figure 6:**
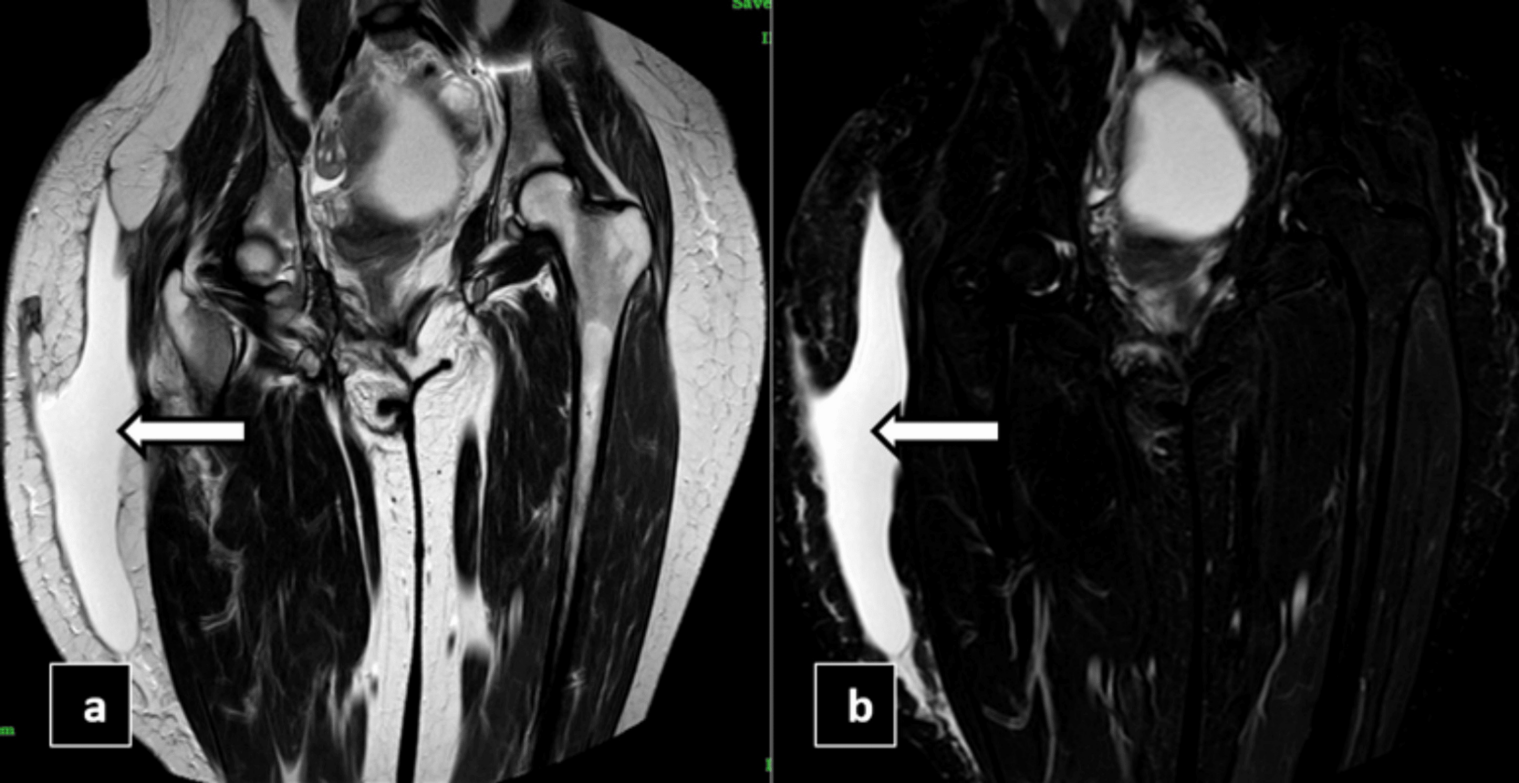
MRI T2 coronal reformatted image (a) and MRI short tau inversion recovery (STIR) coronal reformatted image (b) showing a fairly defined T2 and STIR hyperintense fluid collection noted between the subcutaneous and muscle layers of the right lateral aspect of the thigh region (white arrows).

**Figure 7:**
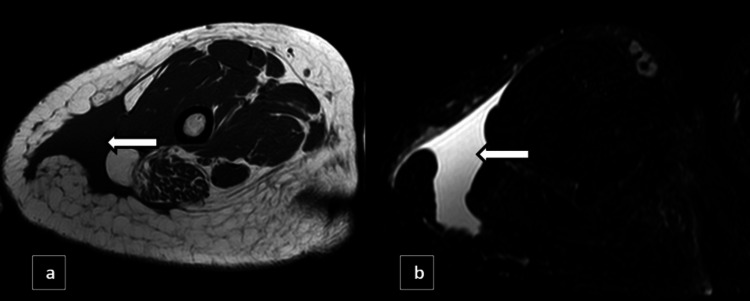
MRI T1 axial reformatted image (a) and MRI short tau inversion recovery (STIR) axial reformatted image (b) showing a fairly defined T1 hypointense and STIR hyperintense fluid collection noted between the subcutaneous and muscle layers of the right lateral aspect of the thigh region (white arrows).

Further imaging with MRI confirmed the presence of a fluid collection in the right lateral thigh region, which appeared T1 hypointense and T2/STIR hyperintense on the scans, as shown in Figures 6-7 suggesting a MLL. Based on these findings, the patient we suggested for aspiration drainage to manage the condition.

Based on these findings, the patient was scheduled for aspiration drainage to manage the condition. The procedure was conducted successfully without any complications, resulting in the complete aspiration of the fluid with sclerodesis and compression bandage for a few weeks. The patient has since recovered well from the procedure.

Case 3

Case 3 was a 51-year-old male who presented to the Outpatient Department (OPD) with chief complaints of intermittent swelling in his right knee, which had persisted for two months following a traumatic incident. He reported that the swelling was non-progressive and fluctuated in intensity, with periods of exacerbation and remission. The patient noted a sensation of warmth over the affected area, which became more pronounced with activity. He described the swelling as worsening during walking and other weight-bearing activities, leading to increased discomfort. Accompanying the swelling, the patient experienced pain localized to the right knee, which varied in intensity and was described as more severe during episodes of swelling. He also reported occasional fevers, which appeared sporadically and were typically associated with the most intense episodes of knee pain and swelling. Due to the chronic nature of the symptoms, their association with trauma, and the presence of intermittent fever, a provisional diagnosis of septic arthritis was considered.

**Figure 8:**
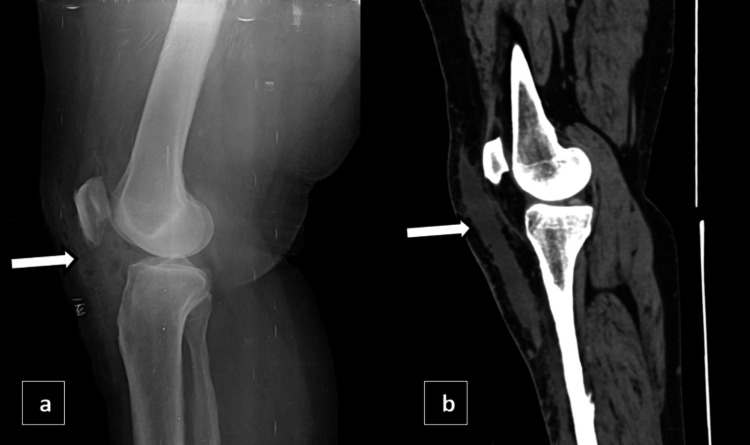
X-ray knee lateral view (a) and CT knee joint sagittal view (b) showing ill-defined haziness in the suprapatellar and infrapatellar regions with the corresponding CT showing a fairly defined hypodense fluid collection between the subcutaneous and intermuscular planes represented by white arrows.

**Figure 9:**
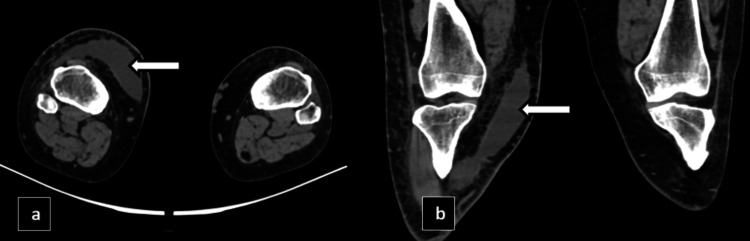
CT axial bilateral knee joint (a) and CT coronal bilateral knee joint (b) showing a fairly defined hypodense fluid collection of volume ~30-40 cc located between the subcutaneous and intermuscular planes represented by white arrows.

X-ray knee and a computed tomography (CT) scan of the right knee was initially performed to provide a detailed assessment of the joint structure and to identify any abnormalities. X-ray showed swelling of the knee joint in the form of ill-defined haziness in the supra and infrapatellar region. The CT scan revealed a fairly defined hypodense fluid collection of volume ~30-40 cc located between the subcutaneous and intermuscular planes, as shown in Figures 8-9.

**Figure 10:**
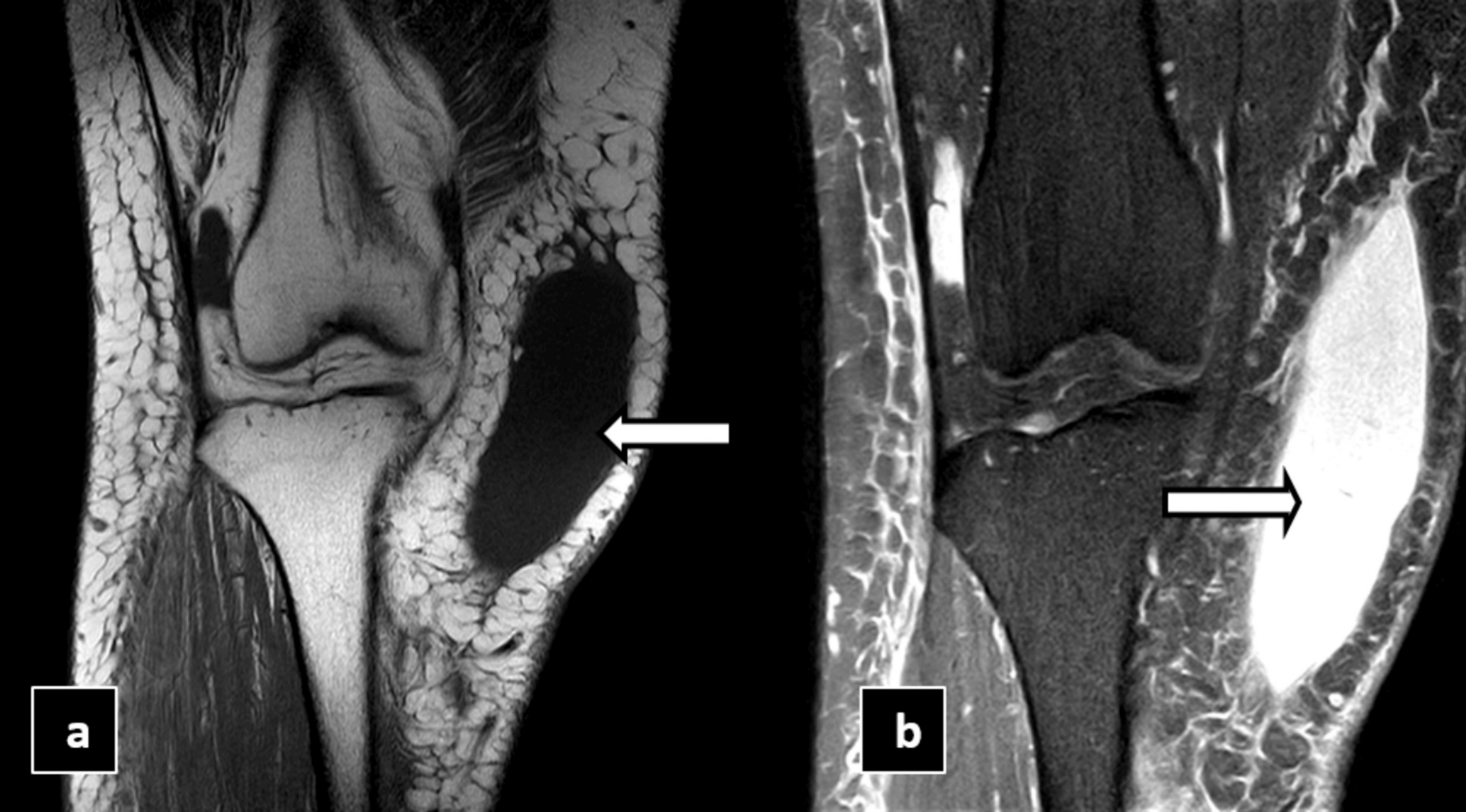
MRI T1 coronal reformatted image (a) and MRI spectral presaturation with inversion recovery (SPIR) coronal reformatted image (b) showing fairly defined T1 hypointense and STIR hyperintense fluid collections between the subcutaneous and muscle layers (white arrows) of the right knee.

To further assess the condition, MRI of the right knee was performed, which showed a fairly defined T1 hypointense and T2/SPIR hyperintense fluid collection noted between the subcutaneous and muscle layers of the right lateral aspect of the thigh region, as shown in Figure 10.

A diagnosis of MLL was made, and the treatment involved the application of compression dressings, maintained for two weeks as part of a "wait and watch" approach. This strategy aimed to reduce the fluid collection through external pressure and to monitor the lesion for signs of resolution or complications. Fortunately, the lesion significantly reduced in size and showed marked improvement during this period.

Case 4

**Figure 11:**
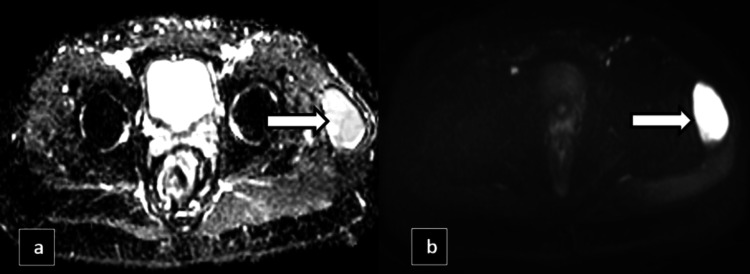
MRI axial reformatted apparent diffusion coefficient (ADC) image and MRI axial reformatted diffusion-weighted imaging (DWI) image (b) showing no true diffusion restriction (hyperintense signals on DWI and ADC) represented by the white arrows.

**Figure 12:**
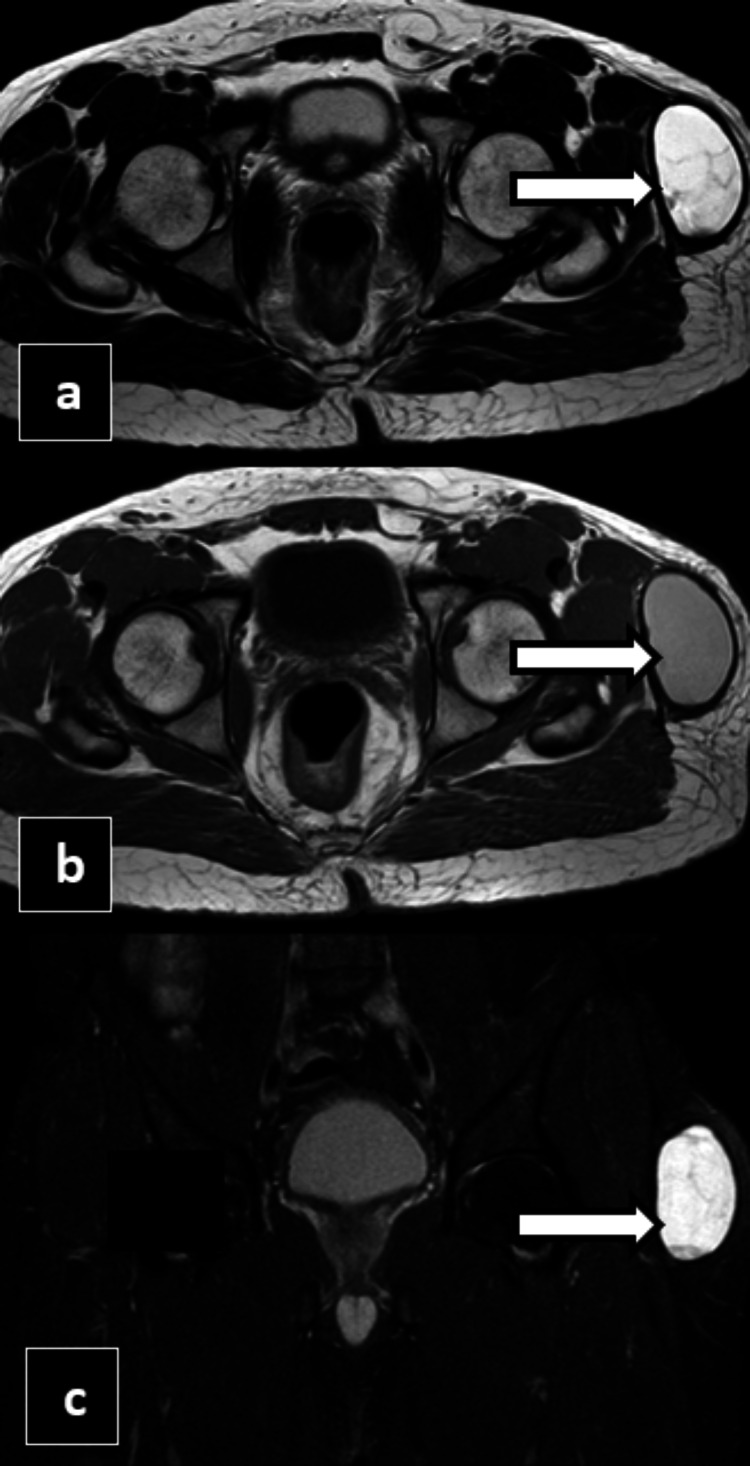
MRI T2 axial reformatted image (a), MRI T1 axial reformatted image (b), and MRI spectral attenuated inversion recovery (SPAIR) coronal reformatted image (c) showing a lentiform-shaped cystic lesion noted in the deep subcutaneous plane, which appears T1 hyperintense and T2/SPAIR hyperintense (white arrows).

In Case 4, a 36-year-old male presented to the Outpatient Department with left hip pain and swelling, following a road traffic accident one month back. He was involved in a collision between his two-wheeler and a four-wheeler, sustaining injuries to his left hip and right leg, along with multiple abrasions over his lower limbs. Since the accident, he reports persistent pain, characterized as a dull ache that worsens with movement and physical activity, and occasional sharp pain during certain movements. The pain slightly improves with rest. There has been no fever or other systemic symptoms reported. He was on conservative management for two weeks, after which the swelling started to increase in size. MRI was done for the patient, and it showed a well-circumscribed oval to lentiform-shaped cystic lesion/collection of volume ~60-70cc noted in the deep subcutaneous plane, which appears T1 hyperintense and T2/SPAAIR hyperintense. No evidence of any true diffusion restriction was noted within the lesion, as shown in Figures 11-12.

A diagnosis of MLL was made, and we suggested USG-guided aspiration, followed by sclerodesis and compression bandage. However, the patient was transferred to his center of preference for the procedure and lost follow-up.

## Discussion

MLLs are rare but significant soft tissue injuries resulting from closed degloving trauma. Characterized by the separation of subcutaneous tissue from the underlying fascia, these lesions create a potential space filled with blood, lymph, and necrotic fat. Initially described in 1853 [1], MLLs continue to pose diagnostic and therapeutic challenges due to their variable presentation and resemblance to other conditions.

Pathophysiology 

The pathophysiology of MLL involves a traumatic shearing force that disrupts the connections between the subdermal plexus and the deeper fascial structures, leading to the accumulation of serosanguineous fluid within the potential space. If left untreated, local inflammation can lead to the formation of a pseudocapsule around the lesion, increasing the risk of complications, such as infection and skin necrosis [1].

Epidemiology 

MLLs are commonly associated with high-energy trauma, particularly in the context of pelvic trauma, where the incidence can reach up to 8.3%. The proximal lateral thigh and peritrochanteric region are the most commonly affected areas due to the high mobility of joints like the hip and knee, coupled with the tough underlying fascia [2,3].

Clinical presentation 

The clinical presentation of MLLs can vary, with localized swelling, fluctuating masses, and pain ranging from dull aches to sharp pain with movement, ecchymosis, and skin hypermobility being common findings. However, the overlap with symptoms of other conditions, such as hematomas and seromas, can lead to misdiagnosis or delayed diagnosis, increasing the risk of complications [4,5].

Imaging features and radiological aspects 

Imaging plays a pivotal role in the diagnosis and management of MLLs. Various modalities can aid in the identification and characterization of these lesions, but magnetic resonance imaging (MRI) remains the gold standard for definitive diagnosis and treatment planning.

*Ultrasonography* 

Point-of-care ultrasonography (POCUS) is a valuable initial tool in the evaluation of MLLs. These lesions typically appear as hypoechoic or anechoic fluid collections located deep to the hypodermis and superficial to the muscular plane. The presence of internal debris or septations within the fluid collection, lacking vascularity on color Doppler imaging, is a characteristic finding. POCUS can aid in early detection, guide percutaneous aspiration or drainage procedures, and monitor the lesion's response to treatment [6].

CT

CT imaging is often employed in the setting of high-energy trauma, where MLLs are commonly encountered. On CT, these lesions appear as well-defined hypodense fluid collections within the subcutaneous or intermuscular planes. CT is particularly useful for evaluating associated injuries, such as fractures, and for measuring the size and extent of the lesion. However, CT's limited soft tissue contrast may restrict its ability to fully characterize the lesion or distinguish it from other fluid collections [7-9].

MRI

MRI is the imaging modality of choice for the definitive diagnosis and characterization of MLLs due to its superior soft tissue contrast and multiplanar capabilities. The imaging appearance of MLLs on MRI varies depending on the stage of the lesion. In the acute phase (within the first week of injury), MLLs typically appear hypointense on T1-weighted images and hyperintense on T2-weighted and fluid-sensitive sequences, such as short tau inversion recovery (STIR) or fat-saturated proton density (FS PD) images. This appearance is attributed to the presence of blood and fluid within the lesion [10-12].

As the lesion progresses to the subacute phase (one to six weeks), the fluid collection becomes more homogeneous and hyperintense on both T1- and T2-weighted images, reflecting the gradual resorption of blood components and the predominance of proteinaceous fluid. In the chronic phase (beyond six weeks), MLLs may develop a peripheral capsule or pseudomembrane, which appears hypointense on both T1- and T2-weighted images, surrounding the hyperintense fluid content. This capsule formation is a hallmark of chronic MLLs and can aid in distinguishing them from other fluid collections [10,11].

Additional MRI sequences, such as diffusion-weighted imaging (DWI) and apparent diffusion coefficient (ADC) mapping, can further characterize MLLs. These lesions typically demonstrate facilitated diffusion, appearing hyperintense on DWI and hyperintense on ADC maps, which can help differentiate them from abscesses or neoplastic lesions [12].

In the literature, six types of MLLs are classified according to their chronicity, tissue composition, and MRI appearance. This classification was proposed by Mellado and Bencardino, who based it on various factors including the shape of the lesion, the presence or absence of a capsule, the signal intensity on T1- and T2-weighted images, and the enhancement pattern observed (Table 1) [12].

**Table 1:** MRI classification of Morel-Lavallée (ML) lesions. Adapted from Ref. [12].

	Type I	Type II	Type III	Type IV	Type V	Type VI
Description	Seroma	Subacute Hematoma	Chronic Organizing Hematoma	Closed Laceration	Pseudo-Nodular	Infected Lesion
Shape	Laminar	Oval	Oval	Linear	Round	Variable Sinus Tract
T1 Signal Intensity	Low	High	Intermediate	Low	Variable	Variable
T2 Signal Intensity	High	High	Heterogeneous	High	Variable	Variable
Capsule Presence	+/-, usually no	Thin	Thick capsule with internal enhancement	Absent	Variable	Thick enhancing capsule

Type I lesions, which account for approximately 60-70% of cases, are described as seromas with a laminar morphology, low T1 signal, high T2 signal, and sometimes a capsule. Type II lesions, making up approximately 10% of cases, are subacute hematomas with an oval morphology, high T1 and T2 signals, and a thin capsule. Type III lesions, comprising 31% of cases, are chronic organizing hematomas with an oval morphology, intermediate T1 signal, heterogeneous T2 signal, and a thick capsule. Type IV lesions are closed lacerations with a linear morphology, low T1 signal, high T2 signal, and no capsule. Type V lesions are pseudo-nodular with a round morphology and variable signals and capsule characteristics. Type VI lesions are infected, featuring a variable sinus tract morphology, variable T1 and T2 signals, and a thick capsule [12,13].

Advanced imaging techniques 

Several advanced imaging techniques have been explored for the evaluation of MLLs, although their routine clinical application remains limited. Ultrasonography with contrast enhancement (CEUS) can aid in the differentiation of MLLs from other soft tissue lesions by demonstrating the lack of intralesional vascularity. Dynamic contrast-enhanced MRI (DCE-MRI) can provide insights into the vascular characteristics of MLLs, potentially aiding in the distinction between acute and chronic lesions. Magnetic resonance neurography (MRN) techniques, such as diffusion tensor imaging (DTI) and tractography, can assess the involvement of adjacent nerves and the extent of nerve compression or displacement by MLLs, which may guide surgical planning [13,14]. 

Imaging in treatment planning and follow-up 

Imaging plays a crucial role in guiding treatment decisions for MLLs. Lesion size is a key factor in determining the appropriate management strategy, with larger lesions (typically >50 cm³) often requiring operative intervention. MRI or CT can accurately measure the lesion's volume and extent, aiding in treatment planning. Imaging is also valuable in the follow-up of treated MLLs. MRI can assess the response to conservative management, such as compression therapy or percutaneous drainage, by monitoring changes in lesion size and signal characteristics over time. In cases of surgical management, imaging can detect residual or recurrent lesions, which may require additional intervention [15,16].

Treatment strategies 

Treatment strategies for MLLs range from nonoperative management, such as compression therapy and percutaneous drainage for smaller lesions, to operative management involving irrigation, debridement, and potential resection of the fibrous capsule in larger or chronic lesions. Single-incision irrigation and debridement (I&D) is recommended for large lesions or those that have failed nonoperative management, with success rates as high as 75%. Dual-incision I&D may be employed when the lesion overlies a surgical approach for fracture management. Open debridement with resection of the fibrous capsule is indicated for chronic MLLs with pseudocyst formation, often requiring multiple surgeries for complete resolution. The clinical management of an MLL involves three phases: acute, subacute, and chronic, with specific treatments tailored to each phase [17].

The clinical management of an MLL involves three phases: acute, subacute, and chronic, with tailored treatments. In the acute phase (less than three weeks), viable subcutaneous tissue is treated with image-guided percutaneous drainage, while non-viable tissue and adjacent injuries require operative debridement and advanced suture techniques. The subacute phase (three weeks to three months) involves drainage, sclerodesis, and compression, with operative capsulectomy for recurrences and antibiotics for infections. The chronic phase (more than three months) follows similar protocols, with additional surgical options such as endoscopic capsulectomy. This approach ensures comprehensive, stage-specific treatment, addressing both immediate and recurring issues promoting optimal healing and recovery. This structured approach ensures comprehensive and stage-specific treatment of MLLs, addressing immediate and recurring issues while promoting optimal healing and recovery [17,18].

Complications and prognosis 

Complications associated with MLL include recurrence, pseudocyst formation, skin necrosis, and perioperative infection. Recurrence is the most common complication, with an incidence of up to 56% in patients managed nonoperatively and 15-20% in those undergoing open debridement. Inadequate debridement and larger lesion size are recognized risk factors for recurrence. Prognosis is influenced by factors such as lesion size, chronicity, and the timeliness of diagnosis and treatment. Smaller, acute lesions diagnosed and managed early tend to have better outcomes, while chronic lesions, particularly those associated with underlying fractures or other complications, may require more extensive surgical interventions and carry a higher risk of recurrence and infection [18].

## Conclusions

MLLs pose significant diagnostic and clinical challenges due to their variable presentation and resemblance to other conditions. Early recognition is crucial for appropriate management and can be achieved through increased clinical awareness and the use of POCUS. Definitive diagnosis with MRI is essential for accurate staging and treatment planning. The development of standardized treatment protocols and further research into MLLs are necessary to improve patient outcomes and reduce the morbidity associated with this condition. Clinicians must be aware of this entity to avoid the mental trauma that patients may experience due to the potential recurrence of these lesions despite aspiration, particularly when the stage of the lesion is not promptly identified. Radiologists play a crucial role in the early detection and accurate diagnosis of MLLs, ultimately contributing to improved patient care and prognosis.
